# Hydrodesulfurization of Dibenzothiophene over Ni-Mo-W Sulfide Catalysts Supported on Sol-Gel Al_2_O_3_-CeO_2_

**DOI:** 10.3390/ma15196780

**Published:** 2022-09-30

**Authors:** Rufino M. Navarro Yerga, Barbara Pawelec, Noelia Mota, Rafael Huirache-Acuña

**Affiliations:** 1 Grupo de Energía y Química Sostenibles, Instituto de Catálisis y Petroleoquímica (ICP), Spanish National Research Council (CSIC), Marie Curie 2, 28049 Madrid, Spain; 2 Facultad de Ingeniería Química, Universidad Michoacana de San Nicolás de Hidalgo, Ciudad Universitaria, Morelia 58060, Mexico

**Keywords:** Al_2_O_3_-CeO_2_ oxides, Ni-Mo-W catalysts, hydrodesulfurization, dibenzothiophene

## Abstract

To achieve sulfur content in gas oil at a near-zero level, new catalysts with improved hydrogenation functions are needed. In this work, new Ni-Mo-Mo hydrodesulfurization (HDS) catalysts supported by Al_2_O_3_-CeO_2_ materials were synthesized to evaluate their efficiency in the reaction of HDS with dibenzothiophene (DBT). Al_2_O_3_-CeO_2_ supports different CeO_2_ loadings (0, 5, 10 and 15 wt.%) and supported NiMoW catalysts were synthesized by sol-gel and impregnation methods, respectively. The physicochemical properties of the supports and catalysts were determined by a variety of techniques (chemical analysis, XRD, N_2_ physisorption, DRS UV-Vis, XPS, and HRTEM). In the DBT HDS reaction carried out in a batch reactor at 320 °C and a H_2_ pressure of 5.5 MPa, the sulfide catalysts showed a dramatic increase in activity with increasing CeO_2_ content in the support. Nearly complete DBT conversion (97%) and enhanced hydrogenation function (HYD) were achieved on the catalyst with the highest CeO_2_ loading. The improved DBT conversion and selectivity towards the hydrogenation products (HYD/DDS ratio = 1.6) of this catalyst were attributed to the combination of the following causes: (i) the positive effect of CeO_2_ in forcing the formation of the onion-shaped Mo(W)S_2_ layers with a large number of active phases, (ii) the inhibition of the formation of the undesired NiAlO_4_ spinel phase, (iii) the appropriate textural properties, (iv) the additional ability for heterolytic dissociation of H_2_ on the CeO_2_ surfaces, and (v) the increase in Brønsted acidity.

## 1. Introduction

Traditionally, catalytic hydrodesulfurization of gas oil is performed using bimetallic Ni(Co)-Mo catalytic systems supported by γ-alumina [1,2]. However, these catalysts are not effective for the hydrodesulfurization of crude oils containing refractory compounds, such as 4,6-dimethyldibenzothiophene (4,6-DMDBT). Due to the steric hindrance of alkyl groups, deep hydrodesulfurization of these compounds requires catalysts with enhanced hydrogenation functions [1,2,3,4,5]. In this regard, recent research focuses on the use of the ternary formulation of the Ni-Mo-W catalyst supported by different materials [6,7,8,9,10,11,12,13,14]. This is due to the well-known hydrogenation properties of Ni and the higher hydrogenation capacity of W with respect to Mo [4], as demonstrated by Ni-Mo-W catalysts supported on alumina and tested in the hydrotreating of vacuum residues [7].

Among the various catalyst supports, the most classical one is γ-alumina. However, there are many other forms of alumina (α, χ, η, δ, κ, θ, and ρ), whose formation depends on the heat treatment of aluminum hydroxide or aluminum oxyhydroxide. Some of them are crystalline compounds, while others are ill-defined amorphous compounds [15]. Among these, the most thermodynamically stable form is the polymorphic α-alumina (corundum) phase, while the η, γ, χ, δ, κ, and θ phases are metastable/transitional phases [16]. Metastable γ-alumina is generally used as a support for industrial HDS catalysts due to its low cost, high specific surface area, defective crystal structure, high surface-to-volume ratio, high pore volume, excellent thermal and chemical stability, low density, high hardness and strength, and easy recovery [15,16,17,18,19,20,21]. To improve the mass transfer capacity of alumina, recent research focuses on hierarchical alumina with one-dimensional (1D) and two-dimensional (2D) nanostructure arrays [19]. 

The main disadvantage of alumina support is that it can produce a large amount of coke due to the presence of a large number of weak Lewis-type acid sites on its surfaces [22,23]. However, there is some literature evidence that alumina prepared by the sol-gel method has a more stable support structure. For example, it was found that Ni catalysts supported on alumina and prepared by sol-gel exhibited higher dispersion than when nickel catalysts are prepared by impregnation on γ-Al_2_O_3_, resulting in higher activity and resistance to carbon deposition [24,25,26]. In addition, alumina itself is not an inactive support, as it can react with the active phases. For example, the formation of strong W-O-Al bonds may occur, preventing the complete sulfidation of WO_3_. Moreover, the high affinity of Ni^2+^ ions to coordinate in the Al^3+^ surface vacancies of the alumina support could lead to the formation of spinel-like NiAl_2_O_4_ structures. As a consequence of the limited availability of Ni ions, their deposition at the edges of MoS_2_ crystallites and the formation of NiMo active phases are also limited [4]. To improve the catalytic activity and decrease the drawbacks of the alumina support, its modification with CeO_2_ has been widely studied for dry/steam reforming of methane, automotive exhaust gas conversion, water shift reactions or hydrogenation of CO_2_ to methanol, dimethyl ether (DME), or methane [27,28,29,30,31,32,33,34,35,36].

In addition, ceria has potential catalytic application in hydrogenation reactions, as demonstrated in the liquid-phase hydrogenation of benzoic acid to benzaldehyde [37], in the partial hydrogenation of alkynes to olefins [38,39,40], and in the hydrogenation of naphthalene [41]. In these reactions, modification of alumina with CeO_2_ changes the structural and electronic properties of the active sites, the metal-support interactions, and the catalyst acidity [41], and modifies the dispersion of the active sites [3,27,29,30] without greatly affecting the specific surface area of the support and pore size distribution [26,27]. 

Despite the successful use of CeO_2_ to catalyze hydrogenation reactions [37,38,39,40,41], the use of Al_2_O_3_-CeO_2_ oxides to support HDS catalysts has received very little attention [6,7,42,43,44]. For example, the impregnation of alumina with ceria precursors has been reported to improve the HDS activity of supported MoS_2_ [42] and metallic Pd catalysts [44]. Similarly, NiMo sulfide catalysts supported on Al_2_O_3_-CeO_2_ mixed oxides prepared by mechano-physical mixing demonstrated higher HDS activity than that of a reference NiMo/Al_2_O_3_ catalyst [43]. Although the sol-gel method has been shown to be the best method to obtain mixed oxides with respect to materials prepared with impregnation [4,5,45,46], there are no reports on the use of one-pot synthesis for the preparation of HDS catalysts supported on Al_2_O_3_-CeO_2_. However, using the sol-gel support preparation method, an increase in catalyst acidity could be expected that could contribute to the increase in HDS activity, as demonstrated by the PtPd/Al_2_O_3_-CeO_2_ catalysts tested in the hydrogenation of naphthalene [41]. In this sense, the effects of various preparation methods were investigated by Fan et al. [34]. It was found that the solid solution structure of the coprecipitated sample enhanced the interfacial interaction of Al_2_O_3_/CeO_2_.

Within this scenario, the objective of this work was to synthesize Al_2_O_3_-CeO_2_ materials with the sol-gel method for their use as support for ternary Ni-Mo-W HDS catalysts. The novelty of this work lies in the use of CeO_2_ for the design of the hydrodesulfurization catalyst, since it is widely known that, in contrast to hardly reducible Al_2_O_3_, CeO_2_ has oxidative properties and a good oxygen storage capacity [43], which are the main reasons for its low attention received for the design of HDS catalysts [6,7,42,43,44]. Therefore, the presented activity results in opening a new perspective for the use of easily reducible oxides, such as CeO_2_, for the design of new catalysts for hydrotreating reactions. The Al_2_O_3_-CeO_2_ supports have been prepared with different CeO_2_ loadings (5, 10, and 15 wt.%) to investigate the effect of the nature and structure of the surface ceria species on the final catalytic behavior of the NiMoW/Al_2_O_3_-CeO_2_ sulfide catalysts. It has been shown that the use of the Al_2_O_3_-CeO_2_ support with the highest amount of CeO_2_ (15 wt.%) not only substantially improved the activity of the catalyst with respect to the Ce-free counterpart, but also enhanced its hydrogenation function, which is necessary for deep HDS. In-depth characterization of the NiMoW/Al_2_O_3_-CeO_2_ catalysts using various techniques clarified the origin of these improvements.

## 2. Materials and Methods

All the reagents used for the support and catalyst synthesis were of analytical grade and were used as received without further purification.

### 2.1. Support Preparation 

**Figure 1:**
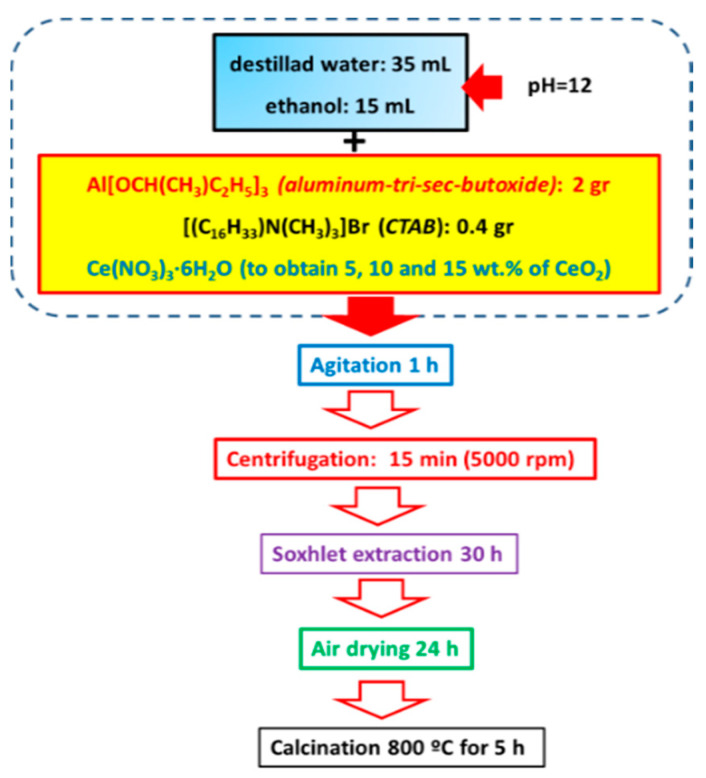
Schematic representation of the γ-Al_2_O_3_-CeO_2_ support synthesis by sol-gel method.

The γ-Al_2_O_3_ was synthesized by the sol-gel method following the method by Zhou et al. [47]. In a standard Al_2_O_3_ support synthesis, 0.4 g of cetyltrimethylammonium bromide (CTAB, Sigma-Aldrich ≥ 98%, St. Louis, MO, USA) was dissolved in 35 mL of deionized water under stirring. The pH value of 12 was obtained by adding NH_4_OH (28–30%, A.C.S. Reagent, J.T. Baker, Santa María Ecatepec, Oaxaca, Mexico). Subsequently, the second solution was prepared by adding, under stirring, 2 g of aluminum tri-sec-butoxide (Al(OC_4_H_9_)_3_; Sigma-Aldrich, 97%, St. Louis, MO, USA) to 15 mL of ethanol. This solution was added dropwise into the surfactant solution under continuous stirring at room temperature for 1 h. After that, the solid was separated by centrifugation (5000 rps) and Soxhlet extraction for 30 h. Next, the as-obtained solid was dried in air at room temperature for 12 h and then treated with He at 800 °C for 5 h to remove the organic template. Synthesis of Al_2_O_3_-CeO_2_ (briefly denoted as Al-Ce(*x*)) with different ceria loadings (5, 10, and 15 wt.%) was in the same manner as it has been mentioned before for the alumina calcined at 800 °C but adding the solution of an appropriate amount of cerium nitrate (Ce(NO_3_)_3_·6H_2_O, 99%, Sigma-Aldrich, St. Louis, MO, USA) dissolved in cetyltrimethylammonium bromide and deionized water. Once the gelation of supports was completed, the solids were dried in air at room temperature overnight and calcined in static air at 800 °C for 5 h. Figure 1 shows a schematic representation of the preparation of the Al_2_O_3_-CeO_2_ materials.

### 2.2. Catalyst Preparation

Ni-Mo-W catalysts supported on Al_2_O_3_ and Al_2_O_3_-Ce(*x*) materials were prepared by incipient wetness co-impregnation using the method described by Mendoza-Nieto et al. [48] using an aqueous solution of heptamolybdate ((NH_4_)_6_Mo_7_O_24_·4H_2_O; A.C.S. Reagent; J.T. Baker; 81.4% MoO_3_; Santa María Ecatepec, Oaxaca, Mexico), ammonium metatungstate ((NH_4_)_6_H_2_W_12_O_40_.xH_2_O; Sigma-Aldrich, ≥66.5% W, St. Louis, MO, USA), and nickel nitrate (Ni(NO_3_)_2_·H_2_O; Sigma-Aldrich, 97%, St. Louis, MO, USA) of precursor salts of Mo, W, and Ni, respectively. The volume of the impregnation solution was adjusted to the pore volume of the support. All supported NiMoW catalysts were prepared with a Ni(Ni+Mo+W) atomic ratio of 0.23. Taking into account the easier Ni-Mo(W) interaction at high pH, the pH of the impregnation solution was first adjusted to pH 12 by adding NH_4_OH (28–30%, A.C.S. Reagent, J.T. Baker; Santa María Ecatepec, Oaxaca, Mexico). Then, citric acid (C_6_H_8_O_7_·H_2_O; Sigma-Aldrich, St. Louis, MO, USA) was added to the impregnation solution in order to stabilize the precursors and avoid precipitation of Ni(OH)_2_ [48]. The impregnated solids were firstly dried in air at 85 °C for 16 h and then calcined in static air at 500 °C for 4 h. The calcined catalysts were stored in a vacuum for further characterization. The catalysts will be denoted hereafter as NiMoW/Al-Ce(*x*) where x is the CeO_2_ nominal content (0, 5, 10 and 15 wt.%).

### 2.3. Catalyst Characterization 

The metal loading of the calcined catalysts was determined by inductively coupled plasma optical emission spectroscopy (ICP-OES) performed with a Perkin Elmer Optima 3300DV (ICP-OES, Perkin Elmer, Waltham, MA, USA). For the analysis, the solids were digested in a mixture of HF, HCl, and HNO_3_ in a microwave oven for 2 h. Aliquots of the solution were then diluted to 50 mL with deionized water (18.2 m quality). The textural properties of the calcined catalysts were determined by N_2_ physisorption at −196 °C with a Micromeritics TriStar 3000 (Micromeritics, Norcross, GA, USA) apparatus. XRD analysis was performed according to the step scanning procedure (step size 0.02° and 0.5 s) with a computerized Seifert XRD 3000P diffractometer (Seifert & Co, Radevormwald, Germany, ), using Ni-filtered Cu Ka radiation (λ = 0.15406 nm) and PW 2200 Bragg-Brentano θ/2θ goniometer. An X-ray photoelectron spectrometer (XPS, VG Escalab 200R, Vacuum Generators, Crowborough, UK) equipped with a hemispherical electron analyzer was used to determine the surface exposure of the Ce^4+^/Ce^3+^ species in Al_2_O_3_-CeO_2_ composites following the method presented by Bêche et al. [49]. The UV-Vis DRS spectra of the samples were recorded on the Varian Cary 5000 UV-Vis (Varian, Santa Clara, CA, USA) spectrometer equipped with an integrating sphere. The bandgap energy (E_g_) was estimated from the relationship between the absorption coefficient and photon energy using Equation (1):

αhυ = (hυ − E_g_)^n^ (1)

where α, h, υ, and E_g_ are the absorbance, Planck constant, photon frequency, photonic bandgap, respectively. The parameter n was assumed to be 2 (indirect electronic transition).

HRTEM images of used catalysts were recorded on a JEOL JEM-3010 (JEOL USA, Peabody, MA, USA) microscope operated at 300 kV with 1.7°A point resolution. The mean particle size and the number of stacked layers of M(W)S_2_ phases were determined by statistical analysis of approximately 250 particles. More details of the analysis techniques employed in this work can be found in [3,4,5].

Diffuse reflectance infrared Fourier transform spectroscopy of adsorbed NH_3_ was employed to investigate the effects of alumina modification with CeO_2_. Spectra were collected on the JASCO FT/IR-6300 (JASCO, Easton, WA, USA) spectrophotometer working at a resolution of 4 cm^−1^ (500 scans), utilizing a Harrick HVC-DRP cell (HVC-DRP cell, Harrick Scientific Products, Pleasentville, NY, USA) that allows in situ treatments with different gases. After sample reduction with pure H_2_ at 450 °C for 306 min, the temperature of the He was cooled down to 120 °C. Prior to spectrum recording, the sample was subjected to the 5 mol.% NH_3_/He analysis gas (Air Liquide) for 45 min. The temperature 120 °C was chosen for NH_3_ adsorption in order to avoid physical adsorption of this molecule.

### 2.4. Activity Tests

The activities of the calcined catalysts were evaluated in the DBT HDS reaction. Sterically unhindered DBT was selected as a model compound due to its abundance in crude oils. Before reaction, the calcined catalysts were activated by ex-situ sulfiding with a 15% H_2_S/H_2_ gas mixture at 400 °C for 4 h. The reaction mixture consisted of 5% vol. of DBT dissolved in decalcification and 0.25 g of DBT dissolved in decaline and 0.25 g of freshly sulfided catalyst. The HDS reaction was carried out in a batch reactor (Parr Instrument Co., Moline, IL, USA) at 320 °C and 5.5 MPa total H_2_ pressure. To exclude external diffusion limitation, the stirring rate was intense (900 rpm). The reaction products were analyzed with an HP 4890 gas chromatograph equipped with a 10-ft packed column with 3% OV-17 as the separation phase in Chromosorb WAW 80/100. Catalytic activity was expressed as total DBT conversion and initial reaction rate (mole of DBT transformed per g of catalyst per second) using the equation described previously [4]. 

## 3. Results

### 3.1. Characterization of Supports

**Figure 2:**
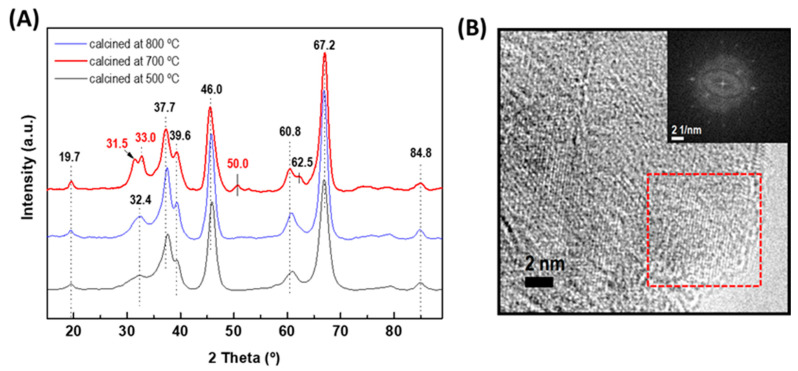
(**A**) XRD patterns of the γ-alumina support calcined at different temperatures (500, 700, and 800 °C). (**B**) HRTEM image of the γ-alumina calcined at 800 °C. The inset shows the fast Fourier transform (FFT) image corresponding to the area marked with a red square in the HRTEM image.

As stated in the introduction part, among the different types of alumina, γ-Al_2_O_3_ is the most widely used support for HDS catalysts. Although the γ-alumina phase could be progressively degraded into α-alumina by heating it at a high temperature, the temperature used for the HDS reaction is much lower than that of its transformation into the α-alumina form (approximately 1000 °C) [15]. Considering that the crystallographic properties of alumina support strongly depend on the temperature of their calcination, pristine alumina was calcined at 500, 700, and 800 °C to select the best calcination temperature for the formation of the γ-Al_2_O_3_ phase (Figure 2A). As expected, the XRD pattern of alumina precursors treated at 500 °C and 700 °C showed peaks at 2θ = 19.7° (very small), 32.4°, 37.7°, 39.6°, 46.0°, 60.8°, and 66.2° (very intense) corresponding to γ-Al_2_O_3_ (JCPDS 86-1410). An increase in temperature from 700 °C to 800 °C led to the splitting of the very broad peak of 32.4° into two peaks (31.5° and 33.0°) and a new peak appeared at 2θ of 50.0°. All these peaks belong to the γ-Al_2_O_3_ phase (JCPDS 00-001-1304). At 800 °C, the transformation of γ-Al_2_O_3_ to α-Al_2_O_3_ did not occur because such a transformation takes place at a temperature much higher than 1100 °C [50]. Therefore, the temperature of 800 °C was selected for the Al_2_O_3_-CeO_2_ support calcination. Figure 2B shows the HRTEM image of the γ-alumina calcined at 800 °C. The fast Fourier transform (FFT) image corresponds to the marked area confirming the formation of γ-alumina. 

**Figure 3:**
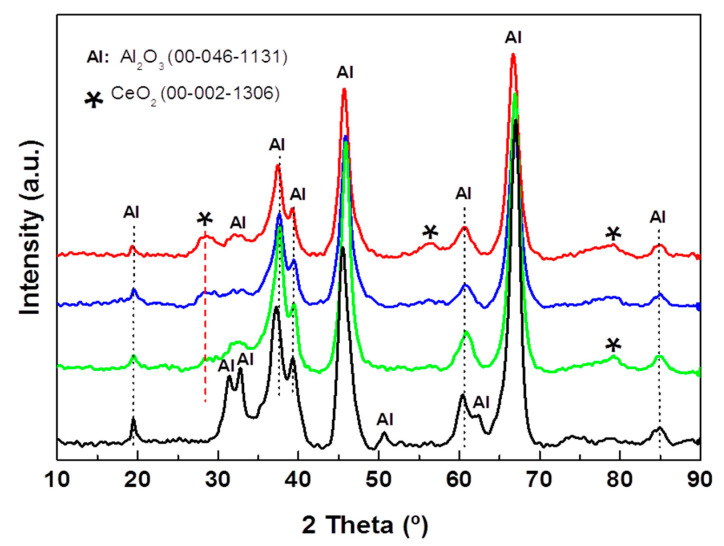
X-ray diffraction patterns of pristine Al-Ce(*x*) supports with different CeO_2_ loadings (*x* = 0, 5, 10, and 15 wt.%).

The effect of CeO_2_ incorporation on the crystallographic properties of the substrate of the alumina supports was investigated by XRD (Figure 3). All the Al_2_O_3_-Cex supports show a diffraction line at 2θ = 28.4° assigned to the (111) crystalline plane of the CeO_2_ phase with a cubic structure (JCPDS 00-002-1306). In addition, Al-Ce15 shows a very broad diffraction line at 2θ = 56.0° corresponding to the (311) crystalline plane of this phase. For all the Al_2_O_3_-Ce(*x*) samples, the peaks are very broad, indicating the formation of small CeO_2_ nanoparticles, and their preferential growth along the (111) direction occurs. Analysis of the 2θ = 28.4° line broadening at half the maximum intensity (FWHM) suggests a decrease in the CeO_2_ crystallite size with an increase in the ceria content, which is in good agreement with that reported for the Al_2_O_3_-CeO_2_ samples prepared by the sol-gel method [41]. 

**Figure 4:**
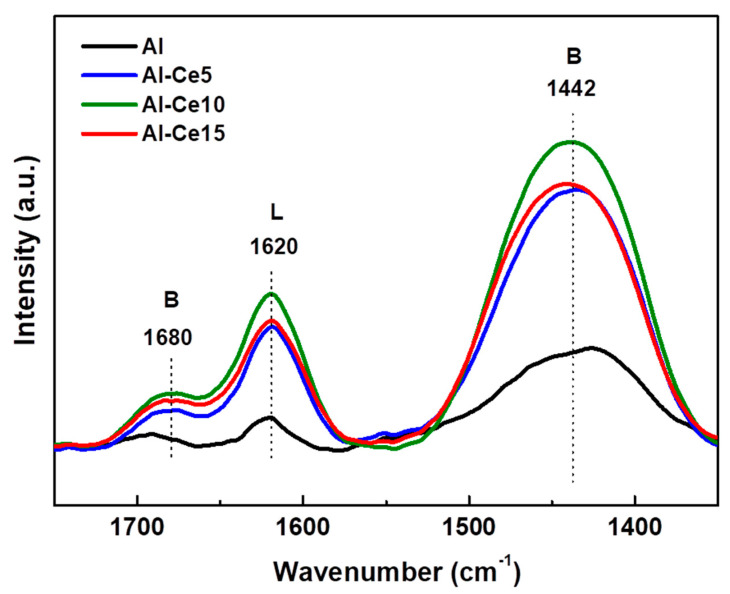
DRIFTS-NH_3_ spectra for pure supports.

The changes in the alumina acidity after modification with varying amounts of CeO_2_ were investigated by the DRIFT spectra of NH_3_ adsorption at 120 °C [23]. Figure 4 shows the DRIFTS-NH_3_ spectra of all supports synthetized with the sol-gel method. The bands at 1680 and 1442 cm^−1^ are due to the δ_sym_(H---N---H) and δ_asym_(H---N---H) vibrations of NH_4_^+^ ions of Brønsted acid sites of the surface, respectively, while the band at 1620 cm^−1^ is commonly attributed to Lewis acid sites [51]. In addition, the observed shadow of approximately 1500 cm^−1^ can be attributed to the bending mode of the surface of the NH_2_ species [52]. Considering the integrated areas of the Brønsted and Lewis bands (data not shown here), both acidities increased after the modification of alumina with CeO_2_. As a consequence, all Ce-containing supports showed a higher Brønsted/Lewis acidity ratio than pure γ-Al_2_O_3_.

The oxidation state of ceria and their surface exposure on Al_2_O_3_-Ce(*x*) supports were investigated with the XPS technique. The core electron levels of Al 2p and Ce 3d are listed in Table 1. The Al 2p and Ce 3d_5/2_ electron levels with binding energies (BE) at 74.5 eV and 881.4 ± 0.1 eV are typical for Al_2_O_3_ and CeO_2_, respectively [30]. The relative Ce^3+^ concentration for the Al-Ce(*x*) mixed oxides was calculated as the peak area of the Ce^3+^ component peak divided by the total peak area of the Ce 3d line. As expected, the amount of total ceria (Ce^4+^ + Ce^3+^) and Ce^3+^ species increases with increasing CeO_2_ content in the mixed oxides (Table 1).

### 3.2. Characterization of the Calcined Catalysts

The Ni, Mo, and W loadings of the synthesized calcined NiMoW/Al-Ce(*x*) catalysts, determined by chemical analysis (ICP-OES technique), are given in Table 2. As can be seen from this table, all the catalysts present a very similar total metal content, ranging from 26.3 wt.% to 27.4 wt.%. It should be noted that the Ce-free catalyst has a little lower Ni promoter content than the Ce-containing catalysts.

**Figure 5:**
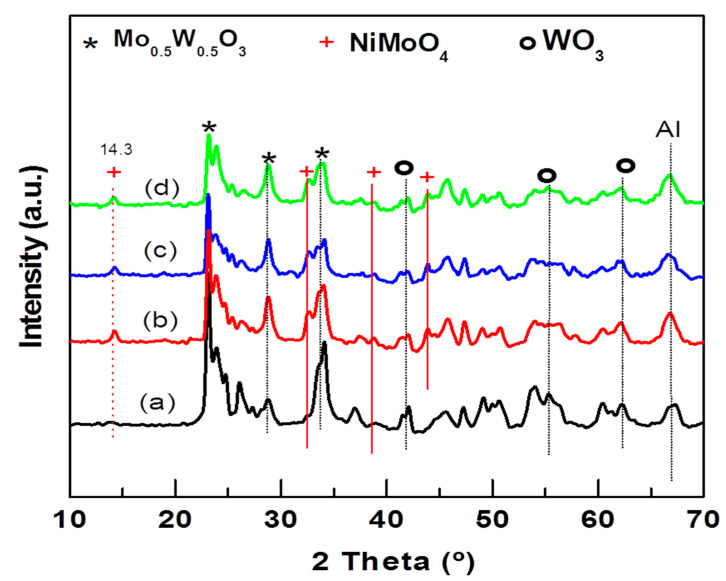
X-ray diffraction patterns of calcined NiMoW/Al and NiMoW/Al-Ce(*x*) catalysts: (**a**) NiMoW/Al; (**b**) NiMoW/Al-Ce5; (**c**) NiMoW/Al-Ce10, and (**d**), NiMoW/Al-Ce15.

The crystallographic properties of the NiMoW/Al-Ce(*x*) calcined catalysts were investigated with the powder XRD technique. The XRD patterns of the calcined catalysts are shown in Figure 5. As expected, all NiMoW/Al-Ce(*x*) calcined catalysts exhibit similar diffraction characteristics to those of the γ-Al_2_O_3_ phase (JCPDS 00-001-1304). In addition, the Ce-containing samples show peaks at 28.3°, 56.4°, and 79.3° that correspond to the CeO_2_ phase (JCPDS 00-002-1306).

XRD patterns of all NiMoW/Al-Ce(*x*) calcined catalysts also exhibit peaks at 2θ = 14.29°, 32.58°, 38.7°, and 43.87° corresponding to the (110), (022), (−132), and (330) planes of crystalline nickel molybdenum oxide (NiMoO_4_; JCDPS 000-033-0948). The diffraction peaks at 23.6°, 33.64°, 41.46°, 54.57°, and 60.25° in 2θ correspond to the (200), (220), (222), (420), and (422) planes, respectively, of the crystalline WoO_3_ phase (JCDPS 00-046-1096). In addition, all calcined catalysts show the peaks characteristic of the Mo_0.5_W_0.5_O_0.3_ phase (JCPDS 00-028-0667). For the Al-Ce(*x*) supported catalysts, the intensity of the NiMoO_4_ peaks increased while those of the WO_3_ and Mo_0.5_W_0.5_O_0.3_ phases decreased with respect to the same peaks observed in the Ce-free sample pattern. The decrease in the peaks corresponds to the WO_3_ and Mo_0.5_W_0.5_O_0.3_ phases, with increasing CeO_2_ content, suggesting that the modification of the alumina with CeO_2_ improves the dispersion of these phases.

Reflections due to the MoO_3_ crystalline phase were not observed in any of the calcined samples, indicating that, if this phase was formed, it either had an amorphous character or its size was below the detection limits of the XRD technique. In this sense, the study by Abello et al. showed that the formation of a 3D phase of MoO_3_ and aluminum molybdate occurs only at high Mo loadings [16]. In the XRD pattern of the NiMoW/Al-Ce(*x*) samples, a shoulder appears at approximately 43°, suggesting the presence of NiO (JCPDS 78-0643). Although crystalline nickel aluminate species were not detected by XRD, their presence cannot be excluded because precise identification of these species is complicated by their proximity to the lines at 2θ = 45.8° and 66.4° typical for γ-Al_2_O_3_ and NiO. However, it could be assumed that the decrease in the peak intensity of approximately 37.1° with increasing CeO_2_ content should be related to the presence of highly dispersed NiO species with some interaction with the Al-Ce(*x*) supports.

**Figure 6:**
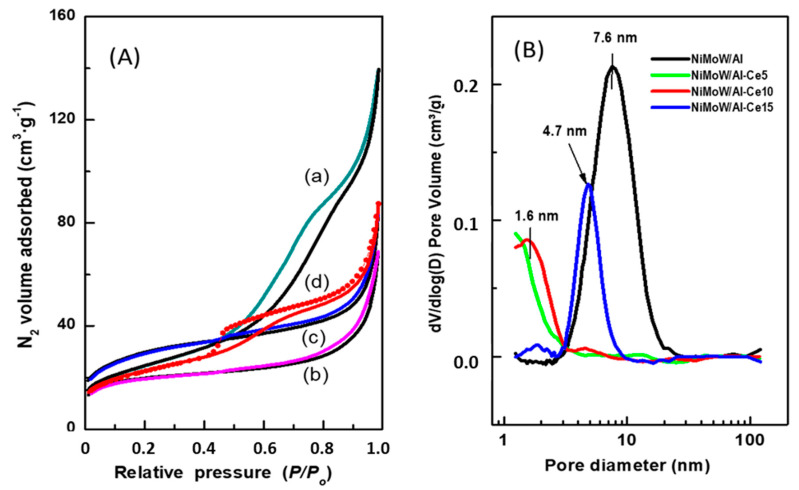
(**A**) N_2_ adsorption-desorption isotherms of the calcined catalysts: (**a**) NiMoW/Al; (**b**) NiMoW/Al-Ce5; (**c**) NiMoW/Al-Ce-10, and (**d**) NiMoW/Al-Ce-15. (**B**) Pore size distribution of the calcined catalysts as determined from the adsorption branch of N_2_ isotherms.

The effect of CeO_2_ loading on the textural properties of the synthesized catalysts was evaluated from the N_2_ adsorption-desorption isotherms (Figure 6A). Considering the IUPAC classification, the adsorption branch of the NiMoW/Al sample is of type IV, which is typical of mesoporous materials. Its hysteresis loop is a combination of H1 and H3 types: the H1-type is typical of mesoporous materials that have nearly cylindrical uniform channels, while the H3-type is characteristic of macroporous materials that have not completely filled pores with pore condensate [53]. On the other hand, the NiMoW/Al-Ce15 catalyst presents an adsorption isotherm with a type IV shape. Its hysteresis loop belongs to the H3 type. It has already been shown [53] that hysteresis appears when an adsorbent has slit-shaped pores. It is noteworthy that the decay of its desorption branch is in a narrow P/P_0_ range and its hysteresis loop starts at a lower relative pressure than that of the sample without Ce, indicating the presence of smaller mesopores. The increase in N_2_ volume observed at a relative pressure above 0.8 is due to a capillary condensation phenomenon. Finally, in contrast to NiMoW/Al and NiMoW/Al-Ce15 catalysts, NiMoW/Al-Ce5 and NiMoW/Al-10 samples show a type II Langmuir isotherm associated with nonporous or macroporous adsorbents [53]. It should be noted that, unlike Ce-free catalysts and NiMoW/Al-Ce15, these catalysts do not possess any hysteresis loop. 

Figure 6B shows the pore size distribution of the calcined catalysts, determined from the adsorption branch of the N_2_ isotherms. As can be seen, the Ce-free sample (NiMoW/Al) has the largest mean pore diameter (7.6 nm) among the catalysts studied, followed by NiMoW/Al-Ce15 (4.7 nm). Notably, the pore size distribution of both samples with a lower Ce content shows a false peak at approximately 1.6 nm due to the tensile strength effect.

The main textural characteristics of the calcined catalysts are listed in Table 3. As expected, the Ce-containing calcined catalysts exhibit smaller total pore volume and pore diameter than the catalysts without Ce, suggesting blockage of the pores by the metal oxides. The incorporation of metal oxides into all supports decreases the specific BET surface area (S_BET_). For example, for the NiMoW/Al catalyst (S_BET_ = 87 m^2^·g^−1^), this decrease was approximately 29% with respect to pristine γ-Al_2_O_3_ calcined at 800 °C (S_BET_ = 122 m^2^·g^−1^). Despite the high decrease in S_BET_, all the calcined catalysts showed high specific BET surface area values in the range of 64–100 m^2^·g^−1^. For both NiMoW/Al-Ce5 and NiMoW/Al-Ce10, the decrease in total pore volume (V_total_) with respect to the NiMoW/Al catalyst cannot reasonably be attributed to the presence of meso- and macropores, as no hysteresis loop is observed for both oxide precursors.

**Figure 7:**
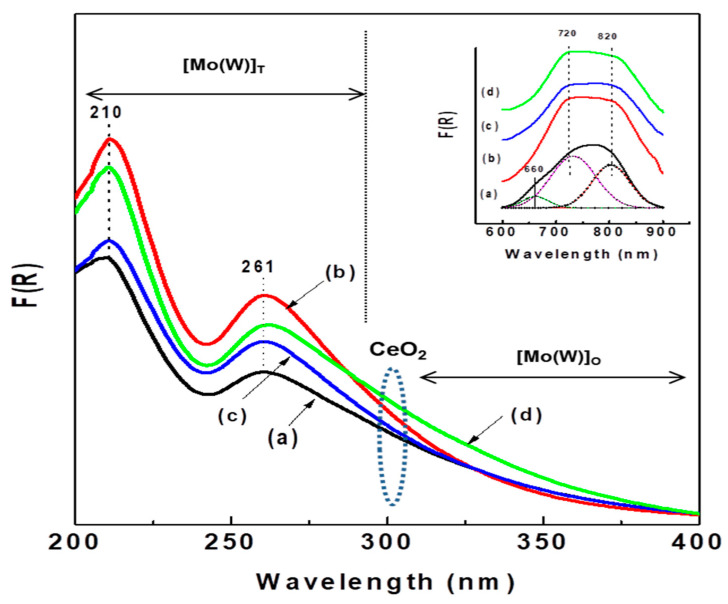
UV-diffuse reflectance spectra of calcined catalysts: (**a**) NiMoW/Al; (**b**) NiMoW/Al-Ce5; (**c**) NiMoW/Al-Ce10, and (**d**) NiMoW/Al-Ce15.

Information on the coordination environment of Ni^2+^ and Mo^6+^(W^6+^) ions in the calcined catalysts was obtained from UV-vis diffuse reflectance spectroscopy. Figure 7 shows the electronic spectra in the 200–400 nm region of all calcined catalysts plotted as a Kubelka–Munk–Schuster function, while the entry in this figure shows their 600–900 nm region. In the 200–400 nm region, all catalysts show two strong bands at approximately 210 and 261 nm, indicating the presence of ligand-metal charge transfer (LMCT); the band at 210 nm is due to the octahedral and/or tetrahedral coordinated Mo(W)^6+^ ions, while the band at approximately 261 nm is assigned to tetrahedral Mo(W)^6+^ species (T_d_) [13]. Considering the intensity of the latter band, the number of tetrahedral Mo(W)^6+^ species follows the trend Al-Ce5 > Al-Ce15 > Al-Ce10 > Al. As expected, all Ce-containing oxide precursors exhibit a shadow at approximately 300 nm due to O^2−^ → Ce^4+^ charge transfer [41,54]. Considering the intensity of this shadow, the amount of CeO_2_ species follows the trend Al-Ce15 > Al-Ce5 > Al-Ce10. Moreover, all catalysts show a large tail between 300 and 400 nm, indicating the presence of octahedrally coordinated Mo(W)^6+^. It is noteworthy that the NiMoW/Al-Ce15 catalyst shows the highest intensity of the absorption band at approximately 325 nm, suggesting that this catalyst might have the highest number of octahedral species among the catalysts studied.

It is well known that CeO_2_ has only octahedral coordination, whereas alumina has both tetrahedral and octahedral coordination. The entry in this figure shows two broad absorption bands at approximately 720 nm and 820 nm (d-d transition) arising from octahedral Ni^2+^ species [55,56]. After Gaussian deconvolution, the sample without Ce shows an additional band at 660 nm, evidencing the presence of a low amount of tetrahedral nickel species, such as the spinel phase NiAl_2_O_4_, which are known to be more difficult to sulfide than octahedral ones. The low amount of those spinel species in the NiMoW/Al-Ce catalysts is probably due to the surface ceria species that induced a decrease in the Ni-alumina interactions. In fact, it should be noted that all NiMoW/Al-Ce catalysts possess mainly octahedral Ni species, which are more reducible than tetrahedral Ni species.

**Figure 8:**
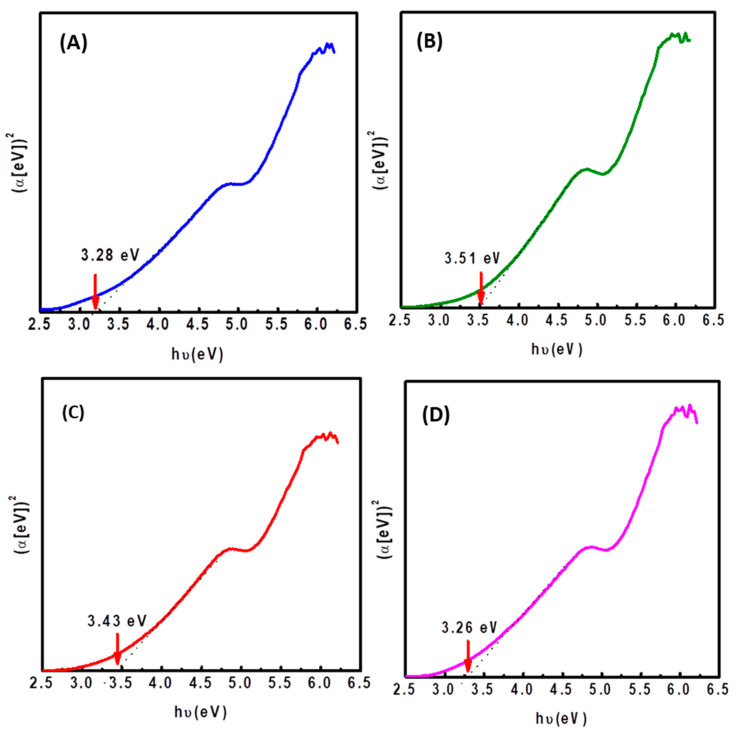
Plot of (αhυ)^2^ vs. photon energy (hυ) for calcined NiMoW/Al-Ce(*x*) catalysts: (**A**), NiMoW/Al; (**B**), NiMoW/Al-Ce5; (**C**), NiMoW/Al-Ce10, and (**D**), NiMoW/Al-Ce15.

Diffuse reflectance measurements were used to estimate the bandgap energy (E_g_) of calcined NiMoW/Al-Ce(*x*) catalysts with different CeO_2_ loadings. Figure 8 shows Tauc plots of (αhυ)^2^ vs. photon energy (hυ) [57]. The bandgap energy (Eg) was estimated from the ratio of the absorption coefficient to the photon energy (Equation (1)) by extrapolating the linear portion of the curve to zero absorbance. The corresponding Eg values were found to be 3.51, 3.43, 3.28, and 3.26 eV for Al-Ce5, Al-Ce10, Al, and Al-Ce15 supported catalysts, respectively. These values indicated that there was a small increase in the estimated bandgap energy for NiMoW/Al-Ce5 and NiMoW/Al-Ce10 compared to NiMoW/Al. In contrast to these catalysts, NiMoW/Al-Ce15 exhibits similar E_g_ to the sample without Ce. Since the structural transformation of WO_3_ from amorphous to crystalline phase causes a decrease in E_g,_ while the opposite trend occurs for the MoO_3_ phase [58], the low E_g_ of NiMoW/Al is clearly due to its higher WoO_3_ loading among the studied catalysts. In the case of NiMoW/Al-Ce15, the explanation for its low E_g_ is more difficult due to its lower W loading and similar Mo loading of both samples (Table 2). It is more likely that the low E_g_ of NiMoW Al-Ce15 is related to the presence of isolated CeO_2_ nanoparticles on the surface of the Al-Ce15 support. 

### 3.3. Catalyst Activity

**Figure 9:**
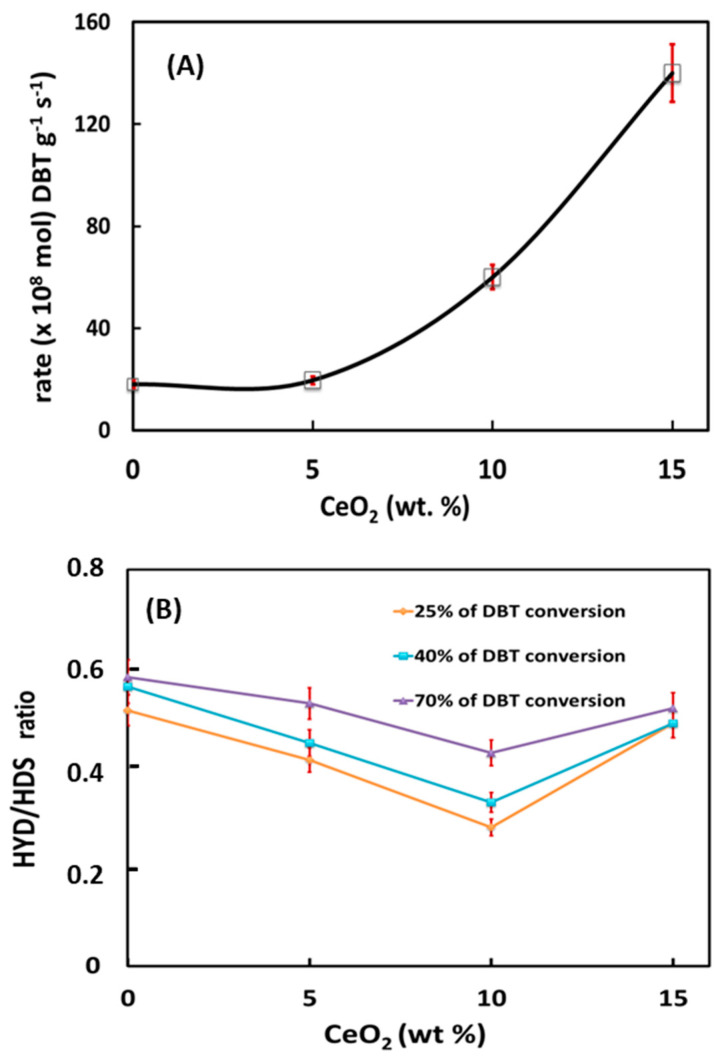
HDS of the DBT reaction (a batch reactor, 350 °C, and 3.1 MPa H_2_ pressure) over NiMoW/Al and NiMoW/Al-Ce(*x*) sulfided catalysts (*x* = 5, 10 and 15 CeO_2_ wt.%): (**A**) initial reaction rates vs. CeO_2_ loading in Al_2_O_3_-CeO_2_ support and (**B**) comparison of the HYD/DDS ratio at the same DBT conversion (ca. 25, 40, and 70%).

Figure 9A presents the initial reaction rates of DBT HDS over sulfided NiMoW/Al and NiMoW/Al-Ce(*x*) catalysts. As can be seen in this figure, the catalyst activity increases gradually as a function of Ce loading in the catalysts, reaching a maximum activity for the catalyst with the highest amount of CeO_2_ (15 wt.%).

Considering the similar metal content of all catalysts, the great improvement of activity with increasing CeO_2_ content clearly indicates the positive effect of CeO_2_ on the HDS activity of sulfided NiMoW/Al-Ce(*x*) catalysts. Our activity results are in line with the study by Kim et al. [59], indicating the higher hydrodesulfurization activity of CoMo/CeO_2_ with respect to CoMo/Al_2_O_3_. 

Table 4 compares the DBT conversion at a reaction time of 5 h of all the synthesized catalysts with two laboratory-fabricated NiMo/γ-Al_2_O_3_ and NiW/γ-Al_2_O_3_ as references. The data presented in this table are of the catalysts that were stabilized for 5 h of reaction. Interestingly, the catalyst modified with 15 wt.% CeO_2_ was stabilized earlier, during the 5-h reaction time, than that supported on alumina (data not shown here), which is in agreement with what has been observed in the literature [35,36]. As can be seen from Table 4, both reference catalysts show lower activity than our NiMoW supported on alumina. Therefore, it could be concluded that the use of our sol-gel alumina as a support together with the use of the ternary NiMoW formulation and higher metal content greatly improved the catalyst performance compared to the bimetallic NiMo and NiW catalysts tested under the same reaction conditions. 

As expected, the products obtained in the hydrodesulfurization of DBT were biphenyl (BP), terahydrodibenzothiophene (THDBT), and cyclohexylbenzene (CHB). BP is formed by the DDS reaction route, while THDBT (intermediate product) and CHB are formed by the HYD reaction route. Since these are parallel routes, the selectivity of the catalysts was calculated as the selectivity ratio HYD/DDS: (CHB+THDBT)/BP. As seen in Figure 9B, at the same DBT conversion of 25, 40, and 70%, all catalysts showed preferential DBT transformation through the direct desulfurization reaction route, with the HYD route being strongly inhibited. However, this situation changes drastically when comparing the HYD/DDS ratio at a reaction time of 5 h. Table 4 compares the HYD/DDS selectivity ratios of the stabilized catalysts with those of the bimetallic reference catalysts tested under the same reaction conditions. As can be observed, the HYD/DDS selectivity ratios at a reaction time of 5 h follow the trend: NiMoW/Al-Ce15 >> NiMoW/Al-Ce10 ≈ NiMoW/Al-Ce5 >> NiMoW/Al. Suddenly, when going from 70 to 98% of the DBT conversion, the most active NiMoW/Al-Ce15 catalyst twists its selectivity and the HYD/DDS ratio changes from 0.6 to 1.7. Notably, this catalyst showed a much higher HYD/DDS selectivity ratio than the reference NiW/ γ-Al_2_O_3_ catalyst (1.7 vs. 1.24). Therefore, the most important finding is related to the improvement of the DBT conversion through the HYD reaction route by modifying the support with a large amount of CeO_2_.

### 3.4. Characterization of Used Catalysts 

**Figure 10:**
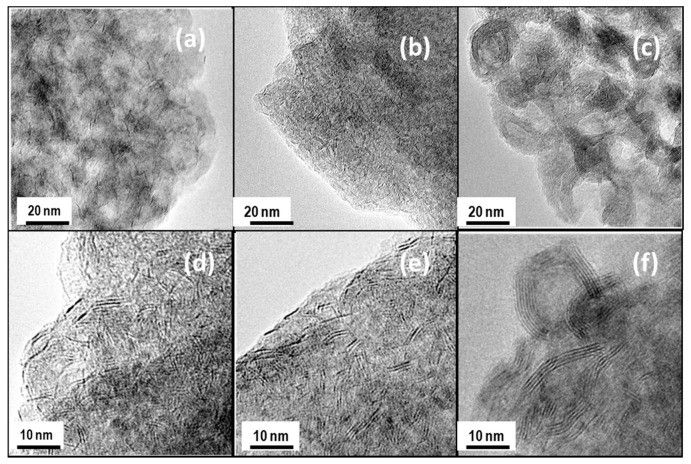
TEM images of used catalysts taken at different scales: NiMoW/Al (**a**,**d**), NiMoW/Al-Ce10 (**b**,**e**), and NiMoW/Al-Ce15 (**c**,**f**).

**Figure 11:**
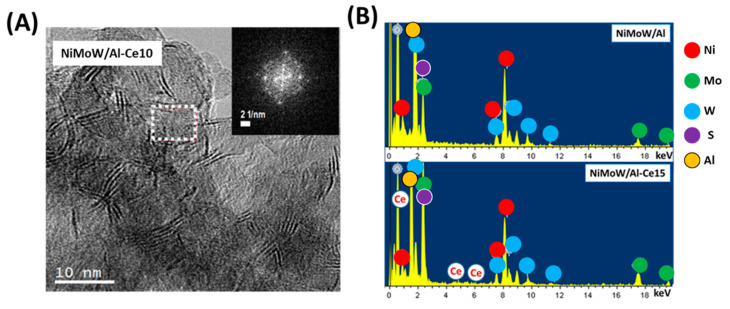
(**A**) HRTEM image of the used NiMoW/Al-Ce10 catalyst. The inset shows the fast Fourier transform (FFT) image corresponding to its selected area showing the cubic symmetry of the CeO_2_ structure. (**B**) EDS spectra of used NiMoW/Al and NiMoW/Al-Ce15 catalysts.

**Figure 12:**
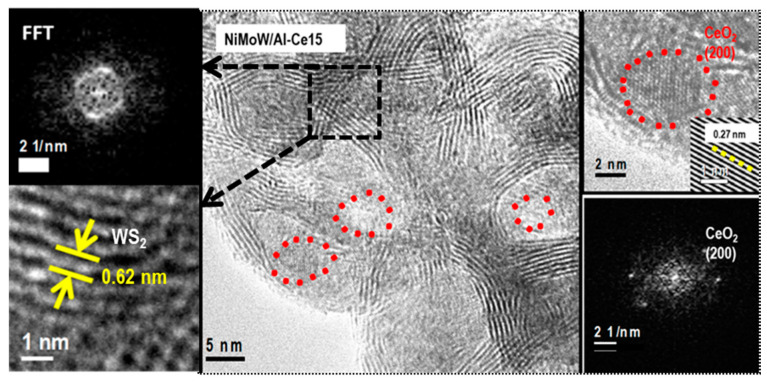
HRTEM image of the used NiMoW/Al-Ce15 catalyst showing the CeO_2_ (red circle) and Mo(W)_2_ (black square) phases. The insets show FFT and IFFT images of the corresponding areas marked in the HRTEM image.

The morphology of the catalysts was used in the DBT HDS reaction and the microstructure of the Mo(W)S_2_ and NiS phases were studied by HRTEM. Representative TEM images of NiMoW/Al, NiMoW/Al-Ce10, and NiMoW/Al-Ce15 are shown in Figure 10, Figure 11 and Figure 12. 

With regard to the active phases, it is well known that Mo(W)S_2_ clusters exhibit a layered structure of Mo(W) atoms located in trigonal prismatic coordination, whereas Ni(Co)-promoted Mo(W)S_2_ layers can be viewed as hexagonally truncated triangles [60]. As can be seen in Figure 10, Figure 11 and Figure 12, all catalysts show the basal plane of the Mo(W)S_2_ layers located parallel to the support surface. As expected, all catalysts show typical fringes corresponding to the (002) plane of the MoS_2_ (0.616 nm) and WS_2_ (0.618 nm) phases. For NiMoW/Al-Ce15, the appearance of the (200) planes of CeO_2_ (d_spacing_ = 0.27 nm, JCPDS 01-075-0390) confirmed the presence of small CeO_2_ crystallites on the surface of the alumina support (Figure 12). The presence of the Ce element on the surface of this catalyst was also confirmed by EDS measurements (Figure 11B). 

**Figure 13:**
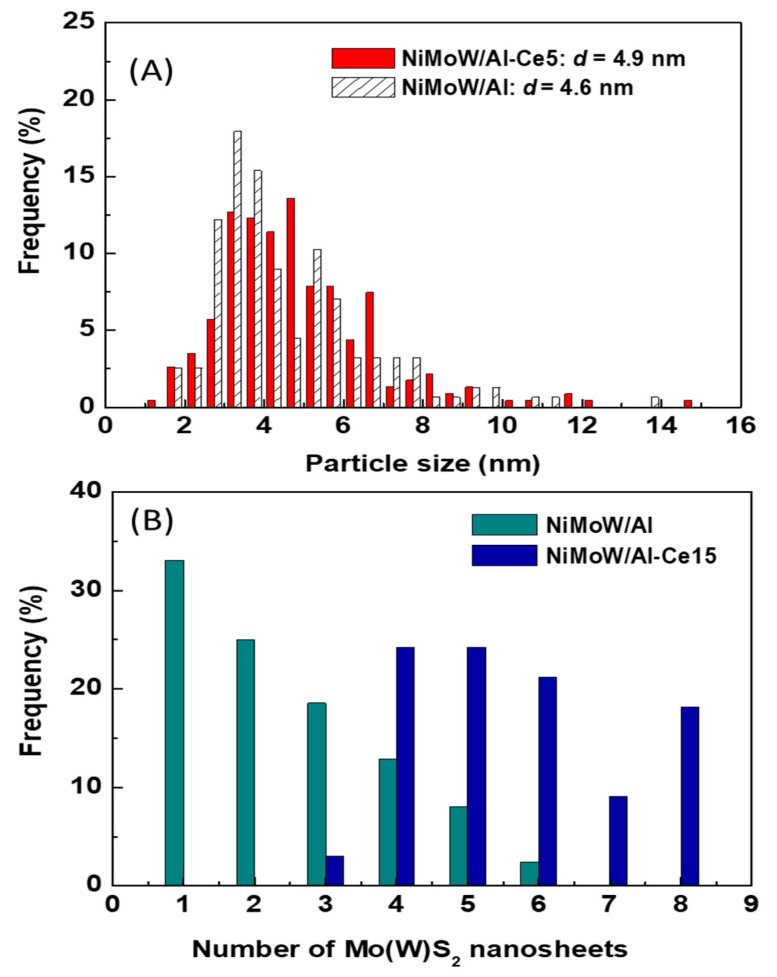
Statistical TEM analysis of the particle size (**A**) and number of Mo(W)S_2_ nanosheets (**B**) of the used catalysts.

For the Ce-free and NiMoW/Al-Ce10 catalysts, statistical evaluation of the Mo(W)S_2_ particle length from various HRTEM images suggests a similar average size of Mo(W)S_2_ nanoparticles (approximately 5 nm) (Figure 13A). Compared to these catalysts, NiMoW/Al-Ce15 shows much larger curved Mo(W)S_2_ sheets with radii of curvature ranging from 1 to 6 nm (Figure 12). To avoid underestimation, no attempt was made to obtain the average particle size of this catalyst. In the case of NiMoW/Al-Ce15, the formation of the onion-shaped Mo(W)S_2_ layers might be induced by the CeO_2_ location along the (002) direction of the Mo(W)S_2_ crystallites. The dislocation of the coplanarity of MoS_2_ planes as a result of the intercalation of Ni atoms in the MoS_2_ interlayer space was reported for unsupported NiMoW catalysts [61]. However, in the case of NiMoW/Al-Ce15, the Ni promoter is probably located at the edge sites of the Mo(W)S_2_ layers, forming “NiMo(W)S” phases. Similar onion-shaped structures were previously observed for NiMo catalysts supported on Ti-modified HMS [8]. The presence of CeO_2_ NPs on the NiMoW/Al-Ce15 surface favors the high stacking of Mo(W)S_2_ layers, as deduced from the comparison of TEM images of the used catalysts in Figure 10. 

Unlike NiMoW/Al-Ce15, NiMoW/Al presents mainly Mo(W)S_2_ phases with 1 and 2 layers (Figure 13B). A similar situation occurs with the NiMoW/Al-Ce10 catalyst (Figure 11A). In the case of the Ce-free catalyst, the growth of large Mo(W)S_2_ particles was avoided, probably because a rougher alumina surface would provide a larger number of nucleation sites, thus, the Mo(W)S_2_ domains would have a lower probability of growing into large clusters [62].

## 4. Discussion

As indicated in the introduction section, new catalysts designed for the hydroprocessing of crude oil should have an improved hydrogenation function. In this regard, this work shows the beneficial effect of alumina modification with a large amount of ceria (15 wt.%) on catalyst morphology and active phase formation.

The disadvantages of alumina-supported reference catalysts with respect to Al_2_O_3_-CeO_2_-supported ones are mainly related to the higher number of active sites and higher Brønsted acidity of the CeO_2_-containing catalysts, as confirmed by HRTEM and DRIFTS-NH_3_, respectively. The latter catalyst characteristic is very important because bifunctional catalysts with both metal and acidic functions are required for the HDS reaction [3,4,5,6,7,8,9,10,11,12,13,14]. However, the linear correlation between catalyst acidity and activity was not observed, indicating that acidity could be a minor factor contributing to the higher activity of Al_2_O_3_-CeO_2_-supported NiMoW catalysts in the HDS reaction.

To explain the observed multiple effects of CeO_2_, we first briefly describe the type of active phases required for DBT transformation via the HYD and DDS reaction routes. In this regard, there is general agreement that the active sites of the metal sulfide catalysts are located at the metal and sulfur edges, as well as at the corners and edge sites of the Mo(W)S_2_ [63]. In Ni-promoted catalysts, the metal atoms at the edge sites of Mo(W)S_2_ layers are replaced by nickel atoms, which under industrial processing conditions have been decorated by hydrogen and sulfur atoms [63]. It is well established that the HDS reaction occurs at the sulfur vacancies of Mo(W)S_2_ crystallites, which are created at the edge sites of these crystallites during the exposure of the catalyst to hydrogen [63,64,65,66]. In this regard, HRTEM characterization is one of the most active NiMoW/Al-Ce15 catalysts, which indicates the formation of onion-shaped Mo(W)S_2_ particles, with larger sizes and stacking layers than its counterpart without Ce. As can be seen in Figure 12, onion-shaped Mo(W)S_2_ layers are formed around the CeO_2_ crystallites located on their supporting surface. These fullerene-like Mo(W)S_2_ clusters exhibit curvature with flat regions separated by strongly localized distorted zones. In these cases, the formation of defective sites in the basal planes is highly possible because the curved layers exhibit weak Mo-S binding strength of the MoS_2_ phase [63]. In the case of the ternary NiMoW catalysts, it can be expected that the Mo(W) atoms at the edges of the Mo(W)S_2_ particles can be partially substituted with Ni atoms. In addition, along the periphery of NiMo(W)S_2_ particles, coordinately unsaturated sites (CUS) can be easily formed at their sulfur and metal edges, corners, and brim sites [63,64,65,66]. The removal of sulfur atoms from the edges of the catalyst particles by molecular hydrogen led to the formation of coordinative unsaturated sites (CUS) that behave as Lewis acid sites, allowing strong binding to the electron-donating DBT molecule [63]. Considering that the most active catalyst was the only one with onion-shaped Mo(W)S_2_ clusters, its improved catalytic behavior suggests the formation of a large number of active sites in the onion-shaped Mo(W)S_2_ phases. The high HDS activity of the NiMoW/Al-Ce15 catalyst is in good agreement with the study by Dayte et al., which related the enhancement of the HDS activity of MoS_2_/TiO_2_ catalysts to the radius of curvature of MoS_2_ layers formed on a rougher surface texture of the TiO_2_ support [62]. Similarly, Berhault and coworkers [67] observed modification of the HDS properties of sulfided CoMo/Al_2_O_3_ catalysts by creating active sites in the curved layers of the MoS_2_ phase. In this regard, for the NiMo/CeO_2_ supported catalyst, Phan et al. observed some Ce-Mo interactions leading to an enhancement of its hydrodeoxygenation activity [68].

**Figure 14:**
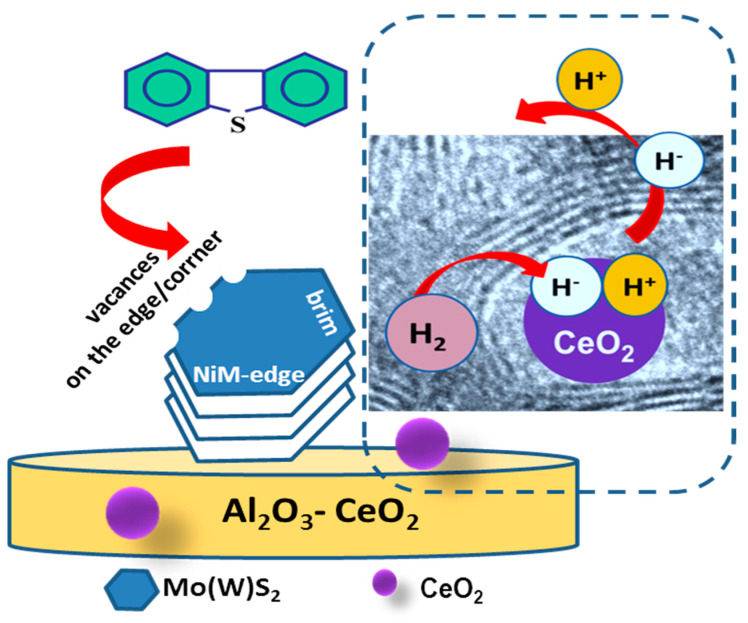
Schematic representation of the DBT HDS reaction mechanism on the surface of the best NiMoW/Al-Ce15 catalyst showing the adsorption of DBT on the CUS sites of the NiMo(W)S phase and the mechanism of the heterolytic dissociation of H_2_ on CeO_2_ nanoparticles.

According to XRD analysis of its oxide precursor, the support surface of this catalyst is decorated by small crystallites of CeO_2_. In this case, the improvement of the hydrogenation function of the NiMoW/Al-Ce15 catalyst can be explained by taking into account the additional ability of the CeO_2_ nanoparticles located on the alumina surface to dissociate H_2_ molecules. In this regard, two mechanisms of H_2_ dissociation on metal oxide surfaces can be taken into account: homolytic dissociation, which produces two hydrogen atoms on two oxygen sites, and heterolytic dissociation, which involves two different adsorption sites: a proton on the oxygen site (H^+^/O^=^) and a hydride (H^−^) on the surface [69]. In this sense, both experimental and density functional theory (DFT) simulations indicated that heterolytic cleavage occurs on difficult-to-reduce Al_2_O_3_ [70], as well as on easily reducible CeO_2_ defective sites [70,71,72,73]. The presence of defective sites on ceria leads to the reduction in ceria and the formation of Ce^3+^ species [70,71,72,73]. Therefore, the sudden change from the direct desulfurization reaction pathway to the hydrogenation pathway can be explained by considering that the high reaction temperature could favor the heterolytic dissociation of hydrogen on the surface of the CeO_2_. A schematic representation of the DBT HDS reaction mechanism on the surface of the best NiMoW/Al-Ce15 catalyst is shown in Figure 14.

Finally, an inhibition of direct desulfurization in the reaction over NiMoW/Al-Ce15 could also be explained considering that the high DBT conversion (97%) leads to the formation of a large amount of H_2_S, which is accumulated in a batch reactor. In such cases, H_2_S adsorbed on the edge of the Mo(W)S_2_ phases inhibits DBT transformation via the direct desulfurization route, favoring its transformation via the hydrogenation route [74]. In fact, there are many literature reports demonstrating that high H_2_S partial pressure severely inhibits the DDS pathway [75,76]. 

In summary, this work demonstrated the positive effect of the formation of onion-shaped Mo(W)S_2_ layers on the catalyst activity. The heterolytic dissociation of H_2_ on CeO_2_ surfaces was proposed as an additional factor that could contribute to an increase in catalyst activity. The enhancement of DBT transformation by the HYD route was linked to the adsorption of H_2_S on the edge sites of the onion-like Mo(W)S_2_ phases.

## 5. Conclusions

In this work, the effectiveness of γ-alumina modification with CeO_2_ on the catalytic behavior of sulfided NiMoW catalysts was evaluated in the liquid-phase DBT HDS reaction. The results show an increase in catalytic activity with increasing CeO_2_ loading, indicating the positive effect of CeO_2_ on the HDS activity of NiMoW/Al-Ce(*x*) catalysts. The high S removal (DBT conversion of 97 wt.%) and the remarkable increase in selectivity towards the hydrogenation route (HYD/DDS ratio = 1.7) were achieved with the best NiMoW/Al-Ce15 catalyst modified with the highest CeO_2_ content (15 wt.%). This catalyst was stabilized more rapidly than that supported on γ-alumina, suggesting the stabilizing effect of the small CeO_2_ nanoparticles decorating the surface of the support. From the catalyst activity-structure correlation, the improved activity of the catalysts supported on Al_2_O_3_-CeO_2_ over those supported only on γ-Al_2_O_3_ was attributed to the formation of the new active phases in the onion-shaped Mo(W)S_2_ layers, to the higher Brønsted acidity, inhibition of NiAlO_4_ spinel phase formation, suitable textural properties, and additional heterolytic dissociation of H_2_ on the surface of CeO_2_ nanoparticles. However, the linear correlation between catalyst acidity and activity was not observed, indicating that acidity could be a minor factor contributing to the higher activity of Al_2_O_3_-CeO_2_-supported NiMoW catalysts in the HDS reaction. The presented results open a new perspective for the use of easily reducible oxides, such as CeO_2_ for the design of new catalysts for hydrotreating reactions.

## Figures and Tables

**Table 1:** Nominal CeO_2_ content and binding energies (eV) of core levels and atomic surface ratios of pristine supports (from XPS).

Support	CeO_2_ (wt.%)	Ce 3d	Al2p	Ce_total_/Al at	Ce^3+^/Al at
Al	-	-	74.5	-	-
Al-Ce5	5	881.5	74.5	0.012	0.0038
Al-Ce10	10	881.4	74.5	0.015	0.0046
Al-Ce15	15	881.3	74.5	0.018	0.0072

**Table 2:** Metal content of calcined NiMoW/Al-Ce(*x*) catalysts (from ICP-OES).

Catalyst	Ni (wt.%)	Mo (wt.%)	W (wt.%)	Total
NiMoW/Al	3.2	8.1	16.1	27.4
NiMoW/Al-Ce5	4.1	5.5	17.0	26.6
NiMoW/Al-Ce10	4.1	5.6	16.9	26.4
NiMoW/Al-Ce15	4.1	5.4	16.8	26.3

**Table 3:** Textural data of calcined catalysts calculated from the N_2_ adsorption-desorption isotherms.

Catalyst	S_BET (m_^2^_·g_^−1^_)_	V_total (m_^3^_·g_^−1^_)_	V_micropores (m_^3^_·g_^−1^_)_	d_mean_ (nm)
NiMoW/Al	87	0.20	0.03	7.6
NiMoW/Al-Ce5	64	0.11	0.02	n.d.
NiMoW/Al-Ce10	100	0.12	0.01	n.d
NiMoW/Al-Ce15	79	0.13	0.01	4.7

**Table 4:** DBT conversions and HYD/DDS ratios of the catalysts stabilized during 5 h of reaction time ^a^.

Catalyst	DBT Conversion (%)	HYD/DDS Ratio
NiMoW/Al	42	0.60
NiMoW/Al-Ce5	56	0.55
NiMoW/Al-Ce10	95	0.65
NiMoW/Al-Ce15	97	1.70
NiW/γ-Al_2_O_3_^b^	69	1.24
NiMo/γ-Al_2_O_3_^b^	59	0.22

^a^ Reaction conditions were: a batch reactor, T = 320 °C, P= 5.5 MPa, reaction time of 5 h. ^b^ Synthetized reference catalysts.

## Data Availability

Not Applicable.
